# Quantitative analysis of the illegal addition of Atenolol in Panax notoginseng based on NIR–MIR spectral data fusion and calibration transfer

**DOI:** 10.1039/d3ra08183d

**Published:** 2024-04-17

**Authors:** Jie Du, Zhengwei Huang, Chun Li, Ling Jiang

**Affiliations:** a Nanjing Forestry University, College of Information Science and Technology Nanjing 210037 China chunli0205@njfu.edu.cn jiangling@njfu.edu.cn

## Abstract

To address the issue of the common illegal addition of Atenolol in Panax notoginseng, we propose an approach that realizes multivariate calibration transfer between different particle sizes based on near-infrared (NIR) and mid-infrared (MIR) spectral data fusion. To achieve high prediction accuracy, we construct three data fusion schemes (full-spectrum fusion, feature-level fusion, and decision-level fusion) that combine NIR and MIR spectral data. Among three data fusion schemes, the feature-level fusion based on the UVE-SPA-PLS model for 120-mesh spectral data achieves optimal prediction accuracy. Here, a Piecewise Direct Standardization (PDS) algorithm has been applied to calibration transfer from 100-mesh and 80-mesh to 120-mesh to reduce the influence of particle size and improve the robustness of the model. The correlation coefficient (*R*^2^) of 100-mesh, and 80-mesh prediction sets can reach 0.9861 and 0.9823, respectively. The corresponding root mean square error (RMSE) are 0.1545 and 0.2045, respectively. This research provides a method for illegal additions in precious herbs and reduces the effect of particle size on spectral modeling, enabling high-precision quantitative detection. In addition, it has important application prospects in reducing experimental losses of precious medicinal materials and ensuring the safe use of Chinese and Western medicines, which provides an alternative method for non-destructive testing.

## Abstract 2

To address the issue of the common illegal addition of Atenolol in Panax notoginseng, we propose an approach that realizes multivariate calibration transfer between different particle sizes based on spectral data fusion.

## Graphical abstract

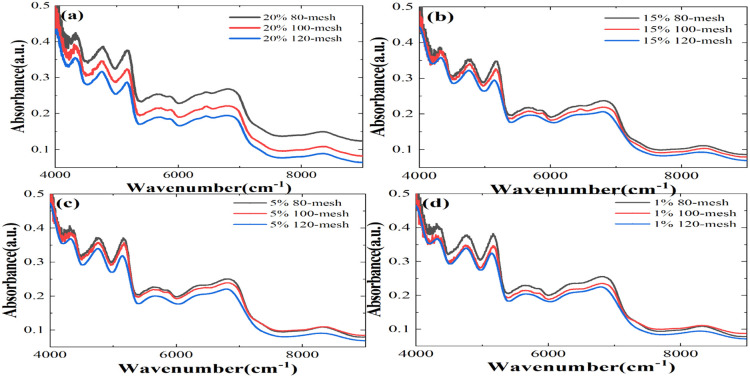

## 1. Introduction

As a precious traditional Chinese medicine (TCM) resource, Panax notoginseng has remarkable efficacy in activating blood circulation, reducing oedema, and enhancing immunity.^1^ Due to the limited geographical areas suitable for its growth, the larger demand for the product in the market has greatly stimulated unscrupulous elements to provide inferior or shoddy products to reap high profits.^2^ For example, anti-hypertensive chemicals, such as Atenolol, and Nifedipine, are directly mixed into Panax notoginseng powder to enhance its anti-hypertensive effect.^3,4^ With the increasing awareness of the health concept, the efficient and accurate quantitative method for analyzing illegally added substances has become a hot research topic in the field of modern medicine and food.

Existing detection methods are mainly based on chemical methods represented by physicochemical tests, gas chromatography, and liquid chromatography.^5^ Although these traditional methods may be reliable, they are limited by the need for complex sample pre-treatment and the inevitable loss of precious TCM. As a fast, non-destructive, and simple technique (only a small amount of samples need to be prepared), spectral analysis technology combined with chemometric methods provides an alternative approach to quality testing of agricultural products and drugs.^6^ Compared to conventional analytical methods, the process of spectral analysis technology has the advantages of rapid, accurate, and non-secondary pollution. Besides, it provides robust analytical reproducibility and cost-effectiveness without compromising the integrity of the sample. Near-infrared spectroscopy (NIR, 700 to 2500 nm) can provide information on the octave and combined-frequency absorption of hydrogen-containing groups (*e.g.*, C–H, O–H, N–H) due to the high penetrating power.^7^ In recent years, NIR has been widely used in multi-component analysis in the areas of food, agriculture, pharmaceutical manufacturing, chemical industry, and biomedicine. Mid-infrared spectroscopy (MIR, 2500 to 25 000 nm), which can effectively provide fundamental frequency vibration information caused by internal vibration and rotational energy level transitions of analyte molecules. It has also been used in analyzing the vibrational modes and chemical bonds of molecules, providing detailed information about the molecular structure.^8^ By correlating the sample spectra and their quality parameters through the calibration model and the spectral information, the quality parameters of the unknown samples can be predicted by machine learning algorithms.^9^ However, quantitative analyses of illegal addition in Panax notoginseng are a complex process. Panax notoginseng usually contains a variety of bioactive components, such as saponins, lactones, and saponic acids.^10^ These components will interfere with the absorption in the spectra, leading to difficulty in the quantitative analysis process. The use of one technique in isolation may not provide sufficient information to enable accurate prediction.

Multi-spectra data fusion achieves resource integration and optimization by merging data from different sources and complementing information between different instruments.^11^ By combining the respective advantages of these spectra, a more accurate and superior prediction model can be obtained.^12^ The basic physical origin of the MIR and NIR are the same. The absorption bands in the infrared spectrum can be viewed as molecular vibration-induced responses. The NIR is primarily an overtone or combined vibration.^13^ However, in the MIR region, absorption is mainly caused by fundamental frequency vibrations, especially the fundamental vibrational leaps of polar groups such as C=O or C–O. In contrast, the signals of these groups are almost absent in the NIR region.^14^ Therefore, it is necessary to fuse the NIR and MIR spectra to obtain more complete information about the analyte, to improve the prediction accuracy of the model.^15^ Spectral information fusion strategies can be classified as full-spectrum fusion, feature-level fusion, and decision-level fusion. Through different data fusion strategies of NIR and MIR, Tao, LY study the process of liquid extraction of various mixtures of two plants, Honeysuckle and *Artemisia annua*. The correlation coefficient (*R*^2^) of the best feature-level data fusion model were improved from 0.900 to 0.984 compared to a single spectral model.^16^ Xinhao Yang *et al.* fused NIR and MIR to quantitatively detect 10-HDA. Compared with the single NIR-model results, the accuracy of the feature-level fusion model is improved from 0.8531 to 0.9585.^17^ These studies mentioned above have proved that multi-spectral information fusion technology can effectively improve the accuracy and stability of the complex analysis model. However, considering the difference in correlation between fusing 2 or more spectra, the optimal fusion strategies requires for further discussion. During the measurement of the spectral data, the applicability and stability of the models are often affected by various multivariate calibration information, such as sample morphology (*e.g.*, particle size), environmental conditions (*e.g.*, temperature), *etc.*^18,19^ As a common form in the pharmaceutical and food fields, solid particles have significant scattering properties in both free powders and solidified compressed forms. This directly results in the impact of particle size parameters on the robustness and accuracy of NIR spectroscopy models.^20,21^ Generally, the smaller the particle size of the analyte, the more stable the corresponding spectral information. To ensure the accuracy of the quantitative analysis model, the Panax notoginseng powder used for measurement needs to be repeatedly sieved to ensure a smaller particle size, which inevitably increases the loss of precious herbs. To solve these problems, Jinrui Mi *et al.* investigated the effect of sample particle size on NIR. A new particle size regression correction (PRC) method was introduced to accurately differentiate three different samples (rice, glutinous rice, and sago).^22^ However, this method usually requires large standard sample volumes and sample pre-treatment and processing are time-consuming and costly.

Based on the similarity of data distribution between different domains, the calibration transfer strategy transfers the trained data model to another related but different data.^23^ Utilizing a set of standard samples from two instruments, this method is commonly used to solve the process differences between different test conditions.^24^ For example, the evaporation of ethanol directly affects the accurate detection of alcohol concentration in high-temperature environments. With the introduction of a calibration transfer model in short-wave NIR (SW-NIR), Barboza *et al.* achieved the same prediction accuracy as 20 °C at 25 °C, 30 °C and 35 °C conditions. The accuracy and stability of the prediction model have been significantly improved, especially at these higher temperatures.^25^ The calibration transfer method can effectively avoid errors caused by different temperatures. Considering the excellent characteristics, model transfer can also be used to reduce the impact of different particle sizes on NIR data. During the modeling process, we further investigate the calibration transfer strategy between different particle sizes based on data fusion strategies to reduce the loss of traditional Chinese medicine in subsequent practical tests.

In this work, we investigate spectral characteristics of mixtures of Atenolol and Panax notoginseng at different concentrations and wavelengths in the NIR and MIR. To further improve the predictive accuracy, we establish three quantitative models using full-spectrum, feature-level, and decision-level fusion methods. After comparing the model results, the best UVE-SPA-PLS dual-band feature fusion model has been selected for further use. To reduce the NIR spectral variability caused by granularity, the PDS method is used for transfer learning with different particle sizes based on feature-level fusion. In the quality inspection of illegally added Panax notoginseng, the model prediction accuracy of this method at 80-mesh and 100-mesh can reach close to 120-mesh. This study provides a comprehensive method for the rapid detection of unreasonable combinations of Chinese and Western medicine and has profound implications for ensuring the safety of medicine dosage.

## 2. Materials and methods

### 2.1 Sample preparation

Atenolol was purchased from Sigma-Aldrich (Sigma-Aldrich Co., St. Louis, MO, USA) and had a purity exceeding 99%. Panax notoginseng was purchased from Nanjing Tongrentang Health Pharmaceutical Group (Nanjing, China) and ground into solid powders. Before sample preparation, all of the materials were dried at 40 °C for 8 hours. The Atenolol was mixed with Panax notoginseng in different proportions. To ensure uniform mixing, we shook mixtures with a shaker for 1 minute. Then the samples were screened sequentially with 80-mesh, 100-mesh, and 120-mesh sieves, with a total of 189 samples. Each mesh has the same 21 different concentrations in which the atenolol concentration ratio increases in the range of 0.5–20%. To avoid the influence of the instrument, each sample had been tested 3 times, and the average of the three measurement results was taken as the final measurement result for the sample.

### 2.2 Spectra acquisition

NIR spectra were collected with the UV-VIS-NIR spectrophotometer (Lambda 950, PerkinElmer, USA). Every spectrum was recorded as the average of 64 scans in the spectral range of 860–2500 nm with 2 nm resolution. FT-MIR spectra were collected with a Frontier FT spectrometer (Vertex 80v, Bruker, USA). All spectra were recorded within the spectral range of 4000–400 cm^−1^ with 4 cm^−1^ resolution, and 16 scans were averaged. Notably, compared to MIR, the operations for NIR are simpler with the mixture placed directly in the module and flattened for direct measurement. In MIR, to minimize variability due to path length in sample preparation with KBr, we use spectral grade purity KBr. In the sample preparation process, we made mixture of 120-mesh samples and KBr in the ratio of 1 : 150. The mass of KBr is fixed and is deducted as background during the tests. The 120-mesh samples and KBr were thoroughly ground in an agate mortar under infrared light. The mixture was then poured into the HF-12 non-removable infrared pressing mould and pressed under a pressure of 15 MPa to make flakes.^26^ In addition, MIR needs compensation operations to eliminate the effects of H_2_O and CO_2_. In large sample measurements, the sample preparation process of NIR has more advantages compared with MIR.

### 2.3 Spectral pre-treatment

The raw spectra obtained from the spectrometer are easily affected by the physical properties of the sample, background information, and noise interference. Optimal pre-processing of the raw spectra can reduce the noise information and effectively extract the key information.^27,28^ Standard Normal Variate (SNV) transformation, Savitzky–Golay (SG),^29^ Multivariate Scatter Correction (MSC) and their combinations are chosen as pre-processing approaches in this study. The SNV and MSC can eliminate the effects of scattering due to uneven particle distribution, thereby enhancing the correlation between spectra and data. However, noise is still present, so the SG smoothing algorithm is used to smooth the spectrum to eliminate high-frequency noise and improve the signal-to-noise ratio. The principle of SG is to fit a least squares polynomial to the data in a moving window. A polynomial of order *k* is synthesised from the data of an odd number of equidistant points in the window to compute a weighted average sum of the points near the centre of the window. It is therefore also known as a polynomial smoothing algorithm. The calculation formula is shown below:1
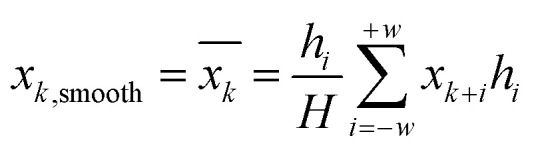
where *h* is the smoothing coefficient, obtained by fitting a polynomial through the least squares method, the coefficient may cut down the misclassification of valid information produced by the smoothing operation, and to some extent make up for its own disadvantage.

By applying the classic Kennard-Stone (KS) uniform sampling algorithm to the NIR, the samples are divided into a 2 : 1 ratio, resulting in 42 samples for the calibration set and 21 samples for the prediction set.

### 2.4 Feature variable extraction

Due to the complexity and high dimensionality of molecular information contained in infrared spectral data, feature selection methods are commonly employed to extract relevant information for the accurate and efficient analysis of complex mixtures. In this study, we mainly use Sparse and informative Partial Least Squares (SiPLS), Successive Projections Algorithm (SPA), and Uninformative Variable Elimination (UVE) for data compression and wavelength selection of the spectral features. The UVE is a commonly used feature wavelength selection algorithm in infrared spectral analysis, aimed at eliminating variables that do not provide useful information.^30^ In particular, when the number of variables is much larger than the number of samples, this method effectively reduces the impact of irrelevant features. The SiPLS algorithm identifies a sparse and informative subset of features highly correlated with the response variables.^31^ The SPA is a forward iterative search method that aims to select spectra with minimal redundancy. It is important to note that during the iteration process, the SPA selects new variables that have the maximum projection onto the previously selected variables, which may result in the exclusion of useful information with smaller projections.^32^ Therefore, a comprehensive consideration needs to be considered when applying these approaches.

### 2.5 Spectral fusion

Based on the fusion structure of multi-spectral data, the fusion strategies can be classified into three categories: full-spectrum fusion, feature-level fusion, and decision-level fusion. After preprocessing, the spectral data from different wavelengths are directly concatenated to form a specific fingerprint of the samples, serving as the input variables for the full-spectrum fusion model. In this study, considering that the MIR and NIR are acquired on different instruments, we normalise the spectral data to avoid disconnections at fusion points. In the feature-level fusion, preprocessed spectral data from different wavelengths are separately subjected to several feature extraction methods (such as UVE, SPA, and SiPLS) to extract informative features. These features are then concatenated into a single feature matrix for multivariate analysis. As it enhances the correlation between the input variables and the substance information in the mixture, feature-level fusion is more effective compared to full-spectrum fusion. In the decision-level fusion, pre-processed spectral data from different wavelength sources are analyzed by separate multi-variate analysis models, and the results from each model are integrated to obtain the fused prediction results at the decision level. In this study, we employ the entropy-weighted TOPSIS voting mechanism, calculating the entropy weight of each spectral model and combining it with the TOPSIS method to compute the optimal and worst distances for each criterion.^33^ This process yields a comprehensive score for each spectral model, determining the weights of each spectral data which can be expressed as:

2 *y*_p-topsis_ = *ny*_NIR_ + *my*_MIR_

where, *y*_*p*−topsis_ represents the predicted values of prediction sets from the TOPSIS. *y*_NIR_ and *y*_MIR_ represent the predicted values of the prediction set in the NIR and MIR regions, respectively. *n* and *m* represent the weights of the NIR and MIR indicators in the TOPSIS calculation.

In addition, we also employ Multiple Linear Regression (MLR) to obtain the integrated results at the decision-level fusion.^34^ The equation for MLR can be expressed as:

3 *y*_p-MLR_ = *b* + *k*_1_*y*_NIR_ + *k*_2_*y*_MIR_

where, *y*_*p*−MLR_ represents the predicted values of prediction sets, obtained from the decision-level data fusion by MLR. *k*_1_ and *k*_2_ represent the coefficients of MLR for the NIR and MIR regions, respectively. *b* is the intercept of the MLR equation.

### 2.6 Calibration transfer based on PDS

Most methods in model transfer for spectral data rely on labeled samples. Labeled sample model transfer algorithms involve establishing a functional relationship between spectra, predicted values, or model parameters obtained from corresponding spectra collected on the host and target machines using labeled standard samples.^35^ In this study, we employ the Piecewise Direct Standardization (PDS) method for the model transfer.^36^ The PDS method utilizes transfer matrices *F*_80_ and *F*_100_ to transform NIR spectra *X*_80s_ and *X*_100s_ (target spectra) into NIR spectra *X*_120m_ (host spectra *X*_80m_ and *X*_100m_). The specific implementation steps of PDS are as follows:

4 *X*_80,*i*_ = [*X*_80s,*i*−*j*_, *X*_80s,*i*−*j*+1_, *X*_80s,*i*+*k*−1_, *X*_80s,*i*+*k*_]

5 *X*_100s,*i*_ = [*X*_100s,*i*−*j*_, *X*_100s,*i*−*j*+1_, *X*_100s,*i*+*k*−1_, *X*_100s,*i*+*k*_]

6 *X*_120,*i*_ = *X*_80,*i*_*F*_80,*i*_

7 *X*_120,*i*_ = *X*_100s,*i*_*F*_100,*i*_

8 *X*_80m,un_ = *X*_80,un_*F*_80_

9 *X*_100m,un_ = *X*_100,un_*F*_100_

where, *X*_120,*i*_ represents the spectral matrix of the standard sample at wavelength point *i* of 120-mesh. *X*_100,*i*_ and *X*_80,*i*_ represent the spectral matrices on both sides of the *i*-th wavelength point with selected window widths of size *k* + *j* + 1. *F*_80,*i*_ and *F*_100,*i*_ represent conversion coefficients of *i*-th wavelength. *F*_80_ and *F*_100_ represent the conversion coefficients of all wavelengths. *X*_80,un_ and *X*_100,un_ represent the spectral matrix of unknown samples at 80-mesh and 100-mesh.

We select the standard sample spectral matrix *X*_120,*i*_ corresponding to the *i*-th wavelength point of the 120-mesh NIR spectrum data from the spectral segments *X*_80s,*k*+*j*+1_ and *X*_100s,*k*+*j*+1_, which are of size *k* + *j* + 1, on both sides of the *i*-th wavelength point in the NIR standard sample spectral matrices *X*_80_ and *X*_100_. These segments form the matrices *X*_80,*i*_ and *X*_100,*i*_, respectively. The *X*_120,*i*_ associated with *X*_80,*i*_ and *X*_100,*i*_. To determine the conversion coefficients *F*_80,*i*_ and *F*_100,*i*_, we use the PLS method. By iterating through *i*, the conversion matrices *F*_80_ and *F*_100_ are computed for all wavelengths within the full spectral range. For achieving transfer spectra consistent with the 120-mesh spectra, the spectra of unknown samples *X*_80,un_ and *X*_100,un_ at 80-mesh and 100-mesh are segmented into optimized window sizes. Through an iterative process, the transfer spectra *X*_80m,un_ and *X*_100m,un_ can be obtained.

## 3. Results and discussion

### 3.1 Spectral data and pre-processing analysis

**Fig. 1:**
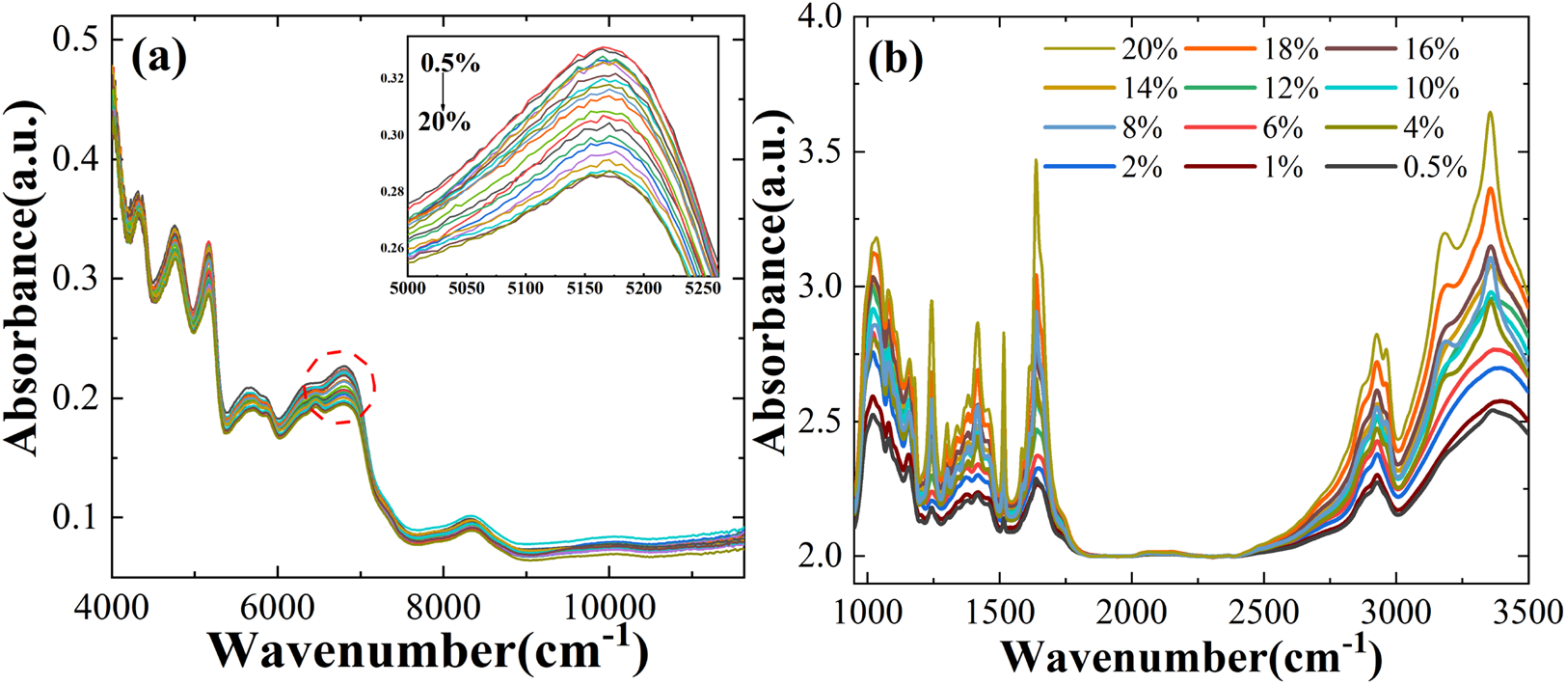
The spectra of the mixture of Atenolol and Panax notoginseng in (a) NIR and (b) MIR.

Fig. 1a and b show the average spectral data between NIR and MIR in which the Atenolol concentration ratio increases in the range of 0.5–20%. Due to the internal molecular vibration of Panax notoginseng, many characteristic peaks can be observed in the wavelength region of 4000–11 627 cm^−1^. From Fig. 1a, it can be seen that the absorbance of the NIR spectra decreases with the increased concentration ratio. There is an obvious negative correlation between the concentration ratio of atenolol and the absorbance of the mixture. As shown in Fig. 1b, similar to NIR spectra, MIR spectra can also be regarded as the fingerprints of the mixture. As the concentration of atenolol increases, the absorbance of the mixture also increases, showing a positive correlation that can be used for further investigation and analysis of the content and interaction between Atenolol and Panax notoginseng in the mixture.

**Fig. 2:**
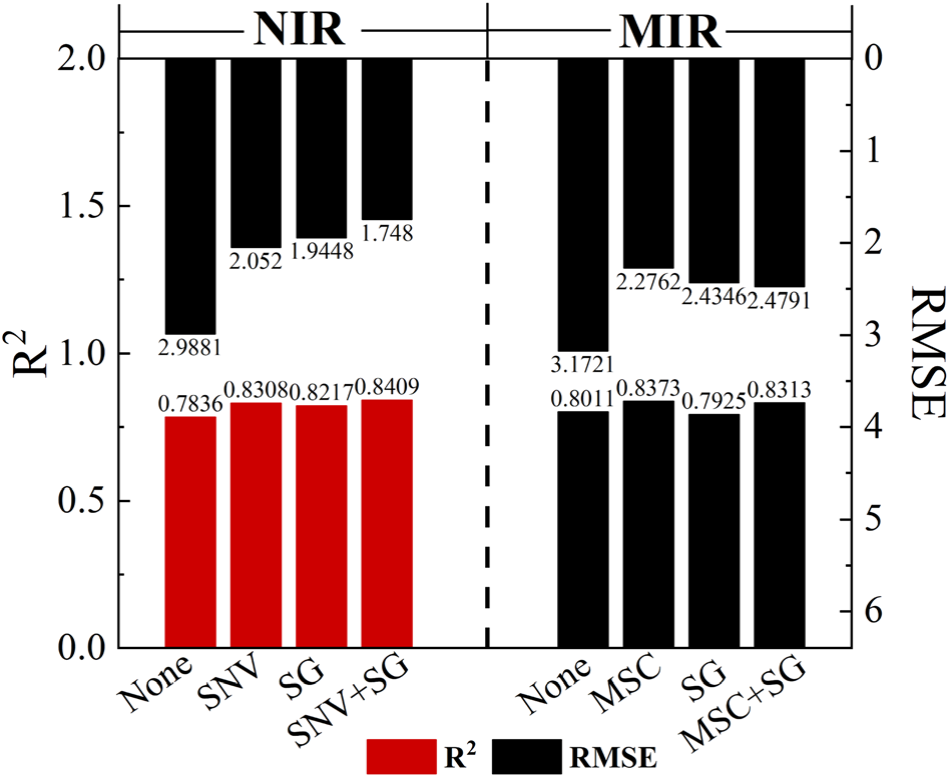
The *R*^2^ and RMSE for pre-processing methods in NIR and MIR.

The raw NIR and MIR spectra contain a lot of information about the chemistry and structure of the sample, but there exists peak overlap and interference from background signals and noise. To improve the signal-to-noise ratio of the spectral data and make the spectral features more obvious, five main methods have been selected for analysis: SG, SNV, MSC, SG + SNV, and SG + MSC. Partial Least Squares (PLS) has been used to predict Atenolol concentrations. In SG, we adopt a window size of 5 and a third degree polynomial. As shown in Fig. 2, through the introduction of pre-processing algorithms, the accuracy of NIR and MIR models can be effectively improved. After the pre-processing with SG + SNV and MSC, the prediction accuracy *R*^2^ of NIR and MIR can be improved to 0.8409 and 0.8373, respectively, improving the correlation between spectral information and the content of the substance.

### 3.2 Quantitative analysis of using spectral fusion

#### 3.2.1. Prediction results using full-spectrum fusion

To further improve the prediction accuracy and compensate for the loss of information caused by single-band modeling, we fuse the spectra of MIR and NIR. Full-spectrum data fusion is the process of concatenating all source data into a single matrix in sampling order. In this study,the fused data is a two-band spectral matrix with a total of 2661 wavelength points.

**Fig. 3:**
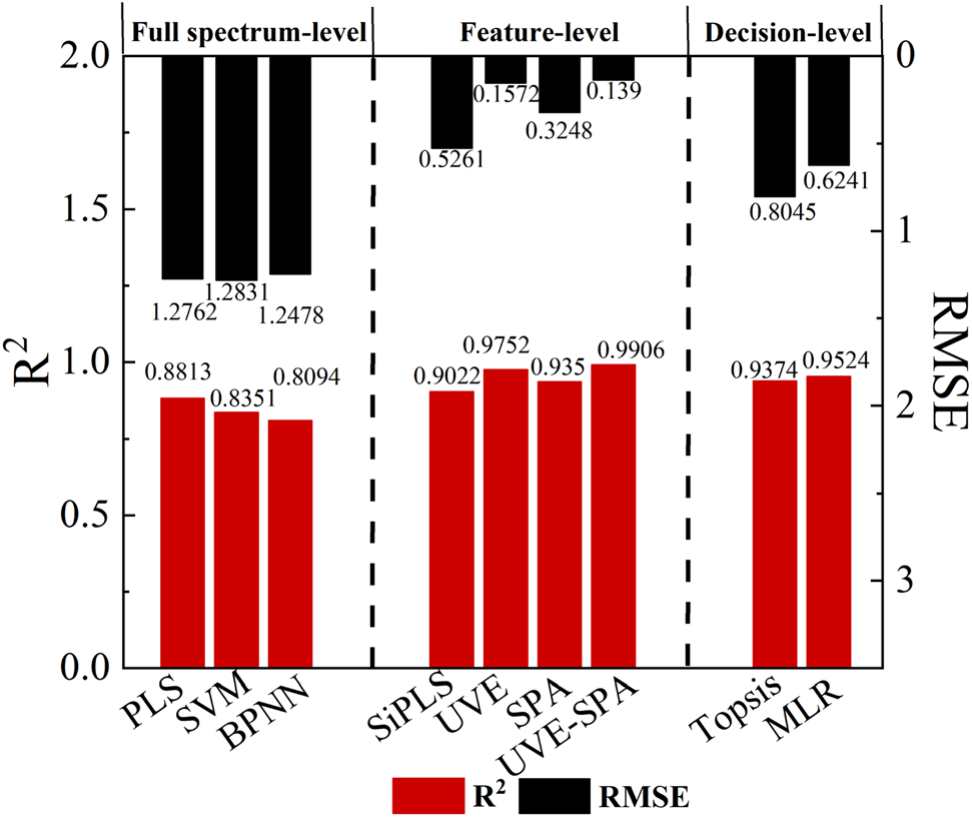
Model construction results of prediction set under different data fusion methods.

We apply the classic Kennard-Stone (KS) uniform sampling algorithm to the NIR and MIR, with a total of 126 samples. Each spectrum has the same 21 different concentrations with 3 samples. The samples are divided into a 4 : 1 ratio, resulting in 101 samples for the calibration set and 25 samples for the prediction set. As shown in Fig. 3, the prediction results of *R*^2^ obtained from PLS, Support Vector Machine (SVM) and Back Propagation Neural Network (BPNN) algorithms can reach 0.8813, 0.8351 and 0.8794, respectively. To avoid over-fitting, the maximum number of latent variables is set to 6 for the PLS model, and the optimal latent variables (LVs) used for each PLS model are determined by the 10-fold cross validation. Based on the PLS prediction model, the *R*^2^ can be improved by 4.80%, and the RMSE can be reduced by 26.99% compared to the single NIR prediction model with higher accuracy. The SVM uses the radial basis function to train the model, with the penalty factor (*c*) set to 5 and the maximum number of iterations set to 100. In BPNN, we mainly focus on three data-type parameters, the number of hidden layers (*l*), the number of hidden neurons (*n*), learning rate (l_*r*_) and a non-data-type parameter transfer function with Tan-sigmoid, *l* = 2, *n* = 6, l_*r*_ = 0.01. The SVM and BPNN prediction models do not show significant improvement in *R*^2^ value due to limited sample size and linearity between Atenolol concentrations and spectral absorbance.

The merging of dual-band spectral data improves the overall quality and richness of data. This allows for better comprehension of the content of the illegal addition of Atenolol in the complex mixture by PLS, SVM, and BPNN. However, this method significantly increases the redundancy of spectral data and the workload of data processing, as well as the complexity of model manipulation.

#### 3.2.2. Prediction results using feature-level fusion

According to previous research, feature-level fusion usually achieves higher accuracy and reliability, and its performance exceeds that of full-spectrum fusion. This approach can extract and integrate the most informative and discriminative features from each source, thereby improving the representativeness of the data. Therefore, we further explore the impact of feature-level fusion on the quantitative analysis of illegally added Atenolol in Panax notoginseng. Feature-level fusion selects features separately from different spectra and combines them into a feature matrix. The extracted feature variables are concatenated into multiple dual-band fused feature matrices. Based on the optimal combination of dual-band fusion feature matrices, the introduced PLS algorithm is used to establish the final fusion model, thereby obtaining the best description of the illegally added Atenolol content in Panax notoginseng.

**Fig. 4:**
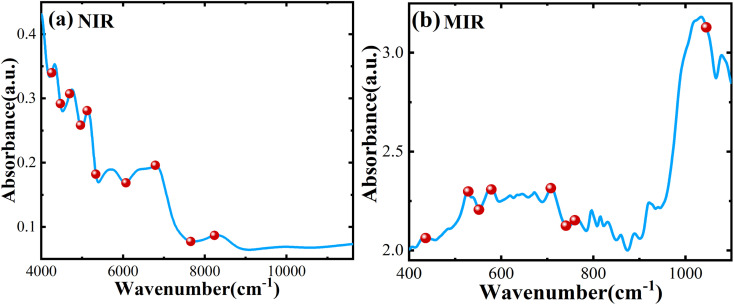
Feature variables after UVE-SPA selection: (a) NIR and (b) MIR.

In this model, we introduce the UVE algorithm to eliminate irrelevant variables. However, during the modeling process, we find that the remaining effective wavelength points are still much larger than the sample size, resulting in high complexity and overfitting of the model. To solve these problems, we use the SPA algorithm to further eliminate redundant information and covariance between variables based on the characteristic wavelength selected by UVE. As shown in Fig. 4a, after the feature extraction operations mentioned above, 10 variables are retained by UVE-SPA in NIR. In Fig. 4b, only 8 variables are selected by UVE-SPA in MIR. The extracted variables contain most of the information in the spectral data, which improves model training efficiency. To ensure the accuracy of the prediction model, we also make a comprehensive comparison of SiPLS, UVE, and SPA feature extraction algorithms. The UVE-SPA feature-level fusion model demonstrates the best prediction potential, as shown in Fig. 3. With the optimal PLS algorithm obtained from fusion results, the *R*^2^ and RMSE of the prediction model can reach 0.9906 and 0.1390, respectively.

It is worth noting that the model established by dual-band feature fusion not only contains more feature information of illegally added Atenolol but also has significant advantages compared with the model obtained from simple data concatenation. Taken together, the UVE-SPA feature extraction method has been utilized to highlight the spectral variables related to the illegal addition of Atenolol.

#### 3.2.3. Prediction results using decision-level fusion

The decision-level fusion approach aims to compensate for the limitations of each model on a single modality by combining the decision outputs of multiple models. Different models can capture different aspects or features of the data, and by integrating this diverse information, a more comprehensive and accurate decision can be obtained. Furthermore, decision-level fusion can increase the robustness of the model, mitigating the impact of misjudgments or erroneous decisions made by a single model. Therefore, we further explore the improvement of constructing a dual-band PLS model using decision-level fusion.

In this study, the SNV-SG and MSC algorithms have been used to pre-process the NIR and MIR spectral data of the doped Panax notoginseng samples. Based on the UVE-SPA algorithm, we perform feature extraction on the processed spectra. Subsequently, the decision-level fusion approach is employed to combine the results of these individual models using the TOPSIS and MLR. The decision-level fusion formula based on TOPSIS and MLR can be calculated with the following equations:

10 *y*_p-topis_ = 0.4073*y*_NIR_ + 0.5927*y*_MIR_

11 *y*_p-MLR_ = 0.3566*y*_NIR_ + 0.6058*y*_MIR_ − 0.0013

It is worth noting that although the decision-level fusion based on MLR achieves higher prediction accuracy (*R*^2^ = 0.9524 and RMSE = 0.6241), it is still significantly insufficient compared with the dual-band feature fusion results, as shown in Fig. 3. Since the decision-level fusion only combines or weights the prediction results of individual NIR and MIR spectra, which results in the information loss. Furthermore, both MIR and NIR originate from the same type of molecular vibrations, the results of NIR and MIR have a certain linear correlation. Therefore, in decision-level fusion, data fusion of NIR and MIR is less advantageous than feature-level fusion.

**Fig. 5:**
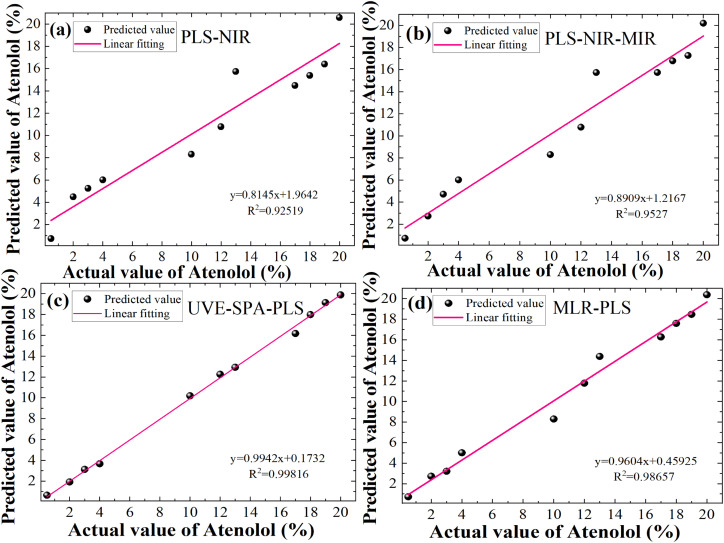
Fitting results between predicted values and actual values of Atenolol: (a) PLS-NIR, (b) PLS-NIR–MIR, (c) UVE-SPA-PLS and (d) MLR-PLS best algorithm for different data fusion strategies.

In summary, we perform a detailed comparison of several quantitative prediction models for the concentration of illegally added Atenolol in Panax notoginseng. The actual and predicted concentration of Atenolol fitting results based on a single 120-mesh NIR with PLS, full-spectrum fusion with PLS, feature-level fusion with UVE-SPA, decision-level fusion with MLR in Fig. 5a–d, respectively. The UVE-SPA-PLS model based on the fusion of the dual-band features of NIR and MIR spectra achieves high-precision quantitative detection, with *R*^2^ of 0.99816. Compared with previous studies using spectral fusion strategy, this study further expands the research scope of spectral fusion strategy in addressing the safety issues of Panax notoginseng.

### 3.3 Calibration transfer based on NIR and MIR spectral data fusion

**Fig. 6:**
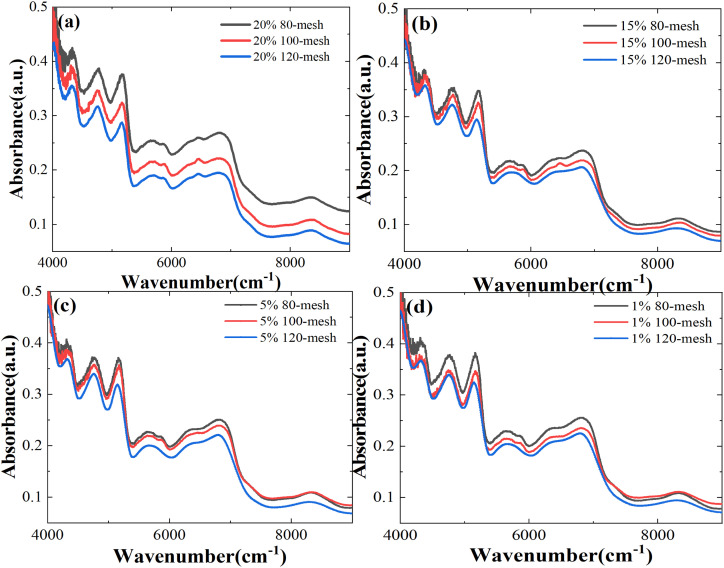
NIR spectra of mixtures of different Atenolol concentrations: (a) 20%, (b) 15%, (c) 5%, and (d) 1% at different particle sizes.

The reduced mesh numbers can effectively avoid the loss of precious medicinal materials during the experiment. However, the larger particle size of Panax notoginseng powder will enhance the scattering effect of NIR transmission spectra in the sample. We measure spectral data for particle sizes of 80 (0.18–0.25 mm), 100 (0.154–0.18 mm), and 120 (0.125–0.154 mm) mesh, as shown in Fig. 6a–d, respectively. With the increased particle size, the NIR absorption spectra of the Panax notoginseng mixtures have been significantly affected at the same concentration, especially in the range of 4000–5000 cm^−1^ wavelength range. The signal-to-noise ratio of spectral data will directly affect the accuracy and stability of the prediction model. In the wavelength range of 5000–9000 cm^−1^, although the NIR absorption spectrum line fluctuates slightly, the overall absorption intensity shifts upward with the increased particle size, which directly leads to the overlap with low-concentration spectral data. An effective method that can avoid the interference caused by the particle size has become an indispensable and important factor in optimizing the quality detection model. Consequently, it is necessary to further utilize chemometrics methods to reduce the effect of granularity on the NIR spectral model and enhance the robustness of the quantitative analysis model.

To quantify the effect of particle size on the NIR model, the PLS algorithm is used to model the NIR spectral data of the 80-mesh and 100-mesh samples. To further explore the impact of particle size on the prediction results, the spectral data at 80-mesh, 100-mesh, and 120-mesh have been used for modeling comparison. As shown in Table 1, the predictive performance of the 80-mesh model is significantly lower than that of the 120-mesh sample under the same spectral scanning conditions. The *R*^2^ and RMSE of the 120-mesh model can reach 0.8409 and 1.7480, while the RMSE of 80-mesh and 100-mesh single NIR models can only reach 1.9445, 1.8921, and the *R*^2^ can reach 0.8313, 0.8362, respectively.

**Table 1:** Results for single and fusion models for different meshes pre- and post-PDS

Mesh	Method	Standard samples	Window width	R^2^	RMSE
120-Mesh	NIR	—	—	0.8409	1.7480
	NIR–MIR	—	—	0.9906	0.139
100-Mesh	NIR	—	—	0.8362	1.8921
	PDS–NIR	3	7	0.8379	1.7563
	NIR–MIR	—	—	0.9879	0.8021
	PDS–NIR–MIR	3	7	0.9861	0.1545
80-Mesh	NIR	—	—	0.8313	1.9445
	PDS–NIR	5	9	0.8336	1.7714
	NIR–MIR	—	—	0.9783	0.9013
	PDS–NIR–MIR	4	9	0.9823	0.2045

Considering the robustness and applicability quantitative analysis model, we use a PDS transfer model to eliminate the effect of particle size in the NIR spectra. In the PDS method used for model transfer, two important parameters (calibration window width and number of standard samples) need to be selected and optimized. During transmission, a small calibration window width will hinder adequate characterization of spectral information between different particle sizes. On the contrary, if the width of the calibration window is too large, it will be necessary to increase the number of standard samples with different particle sizes, thereby increasing the loss of precious medicinal materials. Furthermore, as another important parameter, an insufficient number of standard samples may result in the inability of the transmission matrix to characterize the master and slave spectra accordingly. In the transfer learning process of Panax notoginseng powder particle size, a reasonable selection of standard samples that can effectively reflect the instrumental differences is the key to obtaining the best calibration transfer results.

**Fig. 7:**
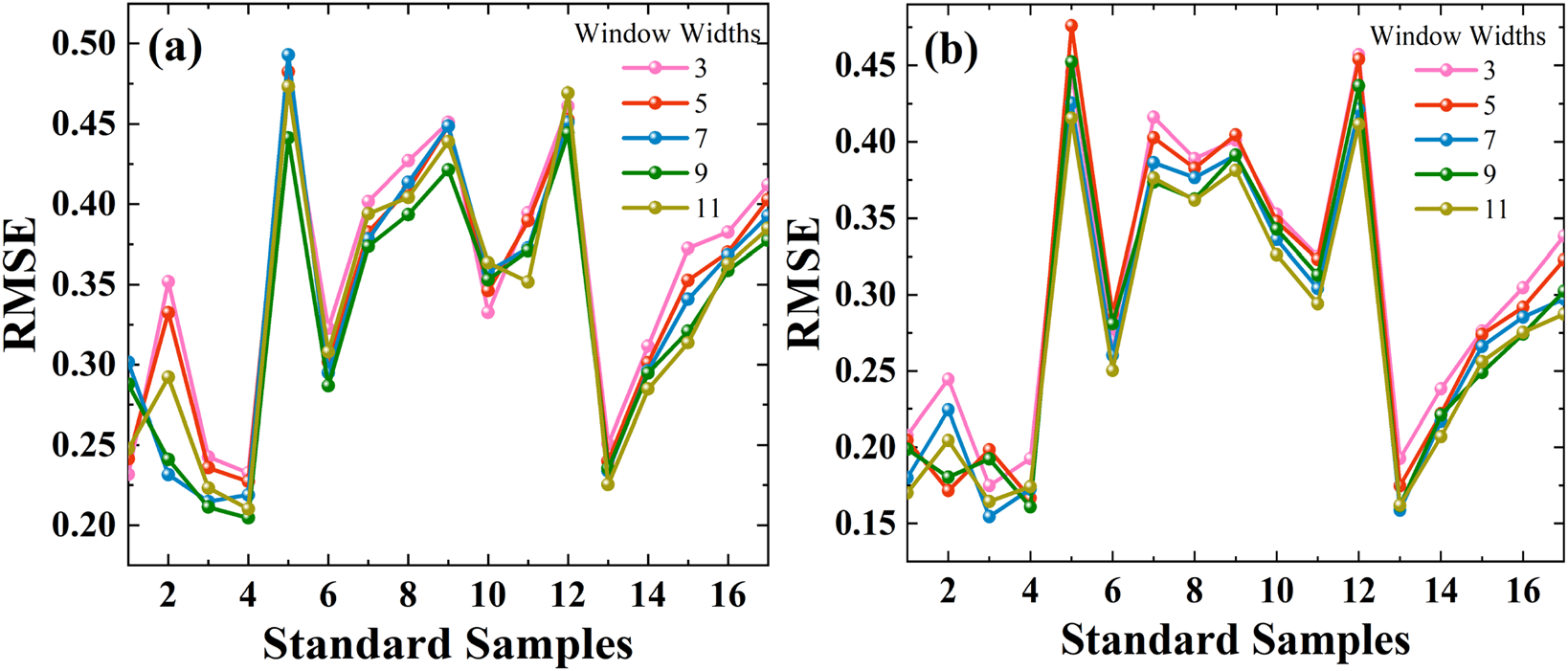
Parameters selection of standard samples and window widths of PDS *via* UVE-SPA-PLS model at different meshes: (a) 80-mesh and (b) 100-mesh.

As shown in Fig. 7a and b, window sizes of 3, 5, 7, 9, and 11 are selected, and 1 to 17 standard samples are chosen from the 80-mesh and 100-mesh calibration sets. By comparing the RMSE, a window width of 9 with 4 standard sample-model yields the minimum RMSE for the 80-mesh NIR spectra data, which are considered the optimal parameters. Similarly, a window width of 7 with 3 standard sample-model yields the minimum RMSE for the 100-mesh NIR spectra data. With the introduced UVE-SPA-PLS model, the prediction accuracies *R*^2^ of the illegally added Atenolol's concentration can be improved by 0.147, 0.1517, and the RMSE can be reduced by 1.0432, 1.09, respectively. Based on the PDS algorithm, the model fusion strategy shows excellent performance when migrating the NIR spectra data of 80-mesh and 100-mesh to 120-mesh. It also improves the prediction accuracy of illegally added Atenolol in Panax notoginseng. The RMSE of the PDS-UVE-SPA-PLS model can be reduced to 0.2045 and 0.1545. The *R*^2^ can reach 0.9823 and 0.9861, respectively. These results confirm that the model transfer combined with the spectral fusion strategy can reduce the interference of the particle size on the NIR spectra, and enable 80-mesh and 100-mesh to achieve high accuracy close to 120-mesh. With the method mentioned above, we can appropriately reduce the particle size requirements in subsequent measurements to reduce the loss of precious herbs. Furthermore, this method can achieve further improvement of the accuracy without the need to repeat the modeling and measure the MIR data of 80-mesh and 100-mesh, ultimately simplifying experimental procedures.

## 4. Conclusions

In this study, for the illegal addition of Atenolol in Panax notoginseng, highly accurate quantitative analysis based on different particle sizes has been realized based on NIR and MIR feature-level fusion strategy combined with PDS calibration transfer. The qualities of infrared spectra have been significantly improved after pre-processed by SNV + SG, and MSC, respectively, which lays the foundation for an accurate analysis. The NIR and MIR spectroscopies are used separately and in combination to estimate the concentration of Atenolol. Three model fusion strategies (full-spectrum fusion with PLS, feature-level fusion with the selected spectral parameters by UVE and SPA, and decision-level fusion with the predicted results by MLR) are discussed. The UVE-SPA-PLS model shows the best performance, achieving the highest *R*^2^ of 0.9906 and the lowest RMSE of 0.139. To reduce the effect of particle size on the NIR model, we use PDS to migrate 80-mesh and 100-mesh into the 120-mesh UVE-SPA-PLS model, while the 120-mesh MIR spectra remain unchanged in fusion model. It effectively improves the prediction accuracy at 80-mesh and 100-mesh particle sizes, respectively. The RMSE of the PDS-UVE-SPA-PLS model can be reduced to 0.2045 and 0.1545, and the *R*^2^ can reach 0.9823 and 0.9861. This study proves that the fusion strategy combined with calibration transfer is a promising method to reduce the interference of the particle size on the NIR spectra and enable 80-mesh and 100-mesh to achieve high accuracy close to 120-mesh. In the subsequent measurement, the requirement for particle size can be appropriately reduced to minimize the loss of valuable medicinal herbs and reduce interference in the detection of other spectra or substances.

## Author contributions

Methodology, investigation, data curation, writing – original draft preparation, Jie Du; writing, review and editing, Zhengwei Huang and Chun Li; conceptualization, supervision, Ling Jiang. All authors have read and agreed to the published version of the manuscript.

## Conflicts of interest

The authors declare no conflict of interest.

## Supplementary Material
